# Narration of a collective traumatic event according to the presence of PTSD and considering the social function

**DOI:** 10.3389/fpsyt.2024.1390470

**Published:** 2024-11-29

**Authors:** Laura Charretier, Francis Eustache, Mickael Laisney, Jacques Dayan, Florence Fraisse, Vincent de La Sayette, Pierre Gagnepain, Amine Chakli, Carine Klein-Peschanski, Denis Peschanski, Peggy Quinette

**Affiliations:** ^1^ Université de Caen Normandie, PSL, EPHE, INSERM, U1077, CHU de Caen, Neuropsychologie et Imagerie de la Mémoire Humaine, GIP Cyceron, Caen, France; ^2^ CHGR Rennes-I, service universitaire de psychiatrie de l’enfant et de l’adolescent, Rennes, France; ^3^ CNRS, Université de Paris 1 Panthéon Sorbonne, EHESS, UMR 8209, European Centre for Sociology and Political Science (CESSP), Paris, France

**Keywords:** posttraumatic stress disorder (PTSD), narratives, social, identity, control

## Abstract

**Introduction:**

Individuals suffering from PTSD recount the traumatic event using perceptual, emotional and sensory details. Memorization and recall of individual events are influenced by the individual’s social function, i.e., what they are willing and able to share about themselves with others and the society. While the influence of PTSD on narratives has been studied, few studies have measured the effect of social function on the link between PTSD and narrative.

**Objectives:**

The aim was to measure trauma narratives of the terrorist attacks of November 13, 2015, according to the presence of PTSD and considering the social function (civil or professional) at the time of exposure.

**Methods:**

Thirty-seven civilians (including 16 women and 21 men) and 22 first responders (including 7 women and 15 men) exposed to the terrorist attacks of November 13, 2015, in France recounted their experiences. Textometric analyses were carried out to characterize the narrative lexicons of the 4 groups (civilians with PTSD; civilians without PTSD, first responders with PTSD; first responders without PTSD).

**Results:**

The narratives of civilians with or without PTSD contain emotional details of the event. The narratives of first responders with and without PTSD contain details of intervention and team. The narratives of civilians and first responders with PTSD contain elements of social and family contacts. The narratives of civilians and first responders without PTSD contains elements of collective control and aid. Civilians with PTSD mostly use the “I” in narratives, while first responders without PTSD mostly use the “we” narrative and the notion of colleague.

**Conclusion:**

Trauma narratives of individuals exposed to the same collective traumatic event who have developed PTSD are characterized by information about social and family contexts. Trauma narratives of individuals without PTSD show a sense of control and collective support. Associated with PTSD, the social function during the event influences the narrative, involving self-centered statements (first person singular, individual reactions) for exposed civilians, and allo-centered statements (colleagues, victims) for exposed professionals. This study underlines the importance of considering the inclusion of reference to the social group to which one belongs when testifying about peritraumatic experiences to others.

## Introduction

On November 13, 2015, simultaneous terrorist attacks targeted the French cities of Paris and Saint-Denis. Claimed by the terrorist organization Daech, these attacks were first perpetrated in the vicinity of the Stade de France during a football match with the explosion of three suicide bombers, then in the 10th and 11th arrondissements of Paris, in which the terrorists opened fire on civilians seated on terraces of Parisian restaurants and cafés and on spectators at a rock concert in the Bataclan concert hall. Emergency services and armed forces quickly arrived, but the death toll was extremely high. A total of 131 people were killed and 413 were hospitalized. The attacks of November 13, 2015, mark a turning point in French civil history. These were indiscriminate attacks, unlike targeted assassinations for political, ideological, or personal reasons (1) and represent the worst terrorist attack on France since the Second World War. The events of November 13, 2015, have increased sense of social cohesion among the French population (2). Before November 13, 25% of those surveyed said they perceived a strong sense of cohesion in French society, compared with 37% afterwards. In addition, a study of the CREDOC (*Research Centre for the Study and Observation of Living Conditions*) showed that in June 2016, a quarter of French people felt they had a personal connection with a victim or witness of the November 13, 2015, attacks, or with one of the locations involved (3). As Truc and colleagues (4) reminds, “*there is no attack without discourse. An attack that doesn’t get people talking about it, that doesn’t elicit statements from political leaders, demands from terrorists, analyses from experts, emotional reactions from citizens, is not an attack: it’s a news item, one homicide among others*”. Fourquet and Mergier (5) showed that in December 2015, 94% of French people said they had discussed the attacks of November 13, 2015, with their loved ones in the days preceding the survey. In June 2016, 93% of French people had a flashbulb memory of the attacks (3), i.e. the feeling of a precise memory of how they became aware of the event and of the identity of the first person they spoke to about the event (6). The highest emotional experiences are shared with others soon after the event (7, 8). Numerous studies have shown the benefit of self-disclosure, i.e. sharing personal and emotional information with others, on resilience and active adaptation to aversive events (9). In the context of a collective event, disclosure increases the sense of belonging to a group and influences the collective memory of the event, i.e. the meaning collectively given to individual representations of the event (10, 11).

The events of November 13 led to post-traumatic stress disorder (PTSD) in 18% of French civilians and 3% of first responders (12). PTSD is a psychological disorder that develops following exposure to an event that may have endangered the life of oneself or others. Among the various traumatic exposures recognized by the DSM-5 (*Diagnostic and statistical manual of mental disorders*, APA, 2013), direct exposure to the traumatic event is one of the most important risk factor for PTSD (13). It challenges the individual’s most basic emotional, physical, and psychological resources.

The potentially traumatic nature of an event can influence the ability to share information with others and the content of this sharing (14–16). Narrative studies are used to gain access to the content of traumatic memories and the way in which traumatized exposed individuals recount, reconstruct, and interpret these memories. Two major literature reviews (17, 18) showed that narratives of individuals with PTSD are dominated by sensory, perceptual, and emotional reactions, which may correspond to a reliving symptom. The intensity of these peritraumatic reactions is frequently singled out as the primary risk factor for the development of PTSD, including peritraumatic dissociation (19, 20). These peritraumatic reactions can result in an altered awareness of the environment and context of the traumatic event (21), notably through peritraumatic dissociation (22–24). However, studies measuring peritraumatic reactions on trauma narratives of PTSD individuals suffer from a lack of standardization in the definitions and methods employed, which prevents the results from being generalized. Narratives analyses also show that linguistic markers linked to mental defeat, death and perceived threat are associated with a greater risk of PTSD. The use of first-person singular pronouns (“I”, “me”, “my”) is also associated with post-traumatic symptoms and chronic PTSD (25–27). But examining the content of traumatic narratives is often decontextualized, as measures fail to consider the influence of individual conditions of traumatic exposure. One function of memory is to inform and update our past, present, and future perceptions of ourselves (28). Given the memory alterations it entails, PTSD may also influence these self-images, and more broadly, self-referential processes (29). Several studies have examined these self-referential processes in trauma narratives but have failed to find consistent results between them (18).

Also, previous narrative studies in PTSD did not measure the influence of the interviewee’s social function on traumatic recounts. Narratives of personal events are stories that form the basis of autobiographical identity and weave moral values directly into identity (30). Narratives are shared with others and depend on the individual’s perception of what is expected of them or the social norms that surround them. Sociologist Erving Goffman speaks of “self-presentation” (31). The individual, in interaction with another, plays a role, maintains a suitable definition of his or her presentation and controls the impression he or she makes. A collective, societal event such as the November 13, 2015, attacks in France affected very different social groups. The attacks perpetrated targeted French civilians, directly exposed to the threat of death and danger, but also the first responders who intervened on the evening of the attacks to bring help and protect civilian victims and French territory. While the study of the traumatic narratives of civilians is now diverse and recognized, having demonstrated the importance of perceptive, sensory, and dissociative cues in them, the study of the narratives of first responders is less prolific. Also, to our knowledge, no study has measured the difference in narrative content that may exist between civilians and first-responders exposed to the same traumatic event, whether they have developed PTSD or not.

First-responders are better prepared for stress and retain greater self-control during the event, a potentially protective control against the development of PTSD. Indeed, peritraumatic reactions are moderated by perceived control over the aversive event (32). Individuals reporting the highest levels of death-related fear and feelings of loss of control during the event were also those reporting the highest levels of peritraumatic dissociation and PTSD (33). “Trained” populations, such as police officers, firefighters, military personnel, or caregivers, are relatively resilient (33–36). In 96 healthy subjects exposed to uncontrollable stress during US Army survival training, Morgan et al. (37) showed that the subjects who were most trained for stress showed reduced levels of peritraumatic reactions. On the other hand, trained populations who had to perform unusual tasks were at greater risk of peritraumatic distress and PTSD (36). Perceived control during the event may protect against PTSD, if the characteristics of the event and its experience do not overwhelm the individual’s resources (38, 39). Professional populations also define their individual identity through strong moral values shared by their social group. Adherence to the group and its values (in this case professional, military, or civic) helps produce a sense of cohesion and facilitates resilience (40). However, social belonging can sometimes be a burden when it comes to recognizing and accepting unwanted emotional or traumatic reactions. According to Maia et al. (41), the lack of voluntary and controlled action induced by peritraumatic reactions in police officers can be seen as cowardice or a moral failing of the professional, who risks stigmatization by his/her peers. In 63 female trauma survivors, Bovin et al. (42) found that the relation between peritraumatic reactions and PTSD is mediated by guilt about passivity during the event. Meyer et al. (43) showed that high self-blame after the event was a significant predictor of PTSD in exposed firefighters.

A systematic review by Bedard-Gilligan & Zoellner (44) shows that the presence of peritraumatic reactions in narratives would depend on individuals’ perceptions of their traumatic experience. If we consider that the traumatic memory is modifiable according to the individual’s values, then the social function during the exposure (civil or professional, for example) and the conditions under which it occurred (peritraumatic reactions and sense of control) can influence the content of the narrative of the experience. However, to our knowledge, no study has considered the effects of social functions and values in the way the traumatic event is recounted by exposed individuals with PTSD compared to exposed individuals without PTSD.

## Present study

The aim of our study was to measure the effect of PTSD on the content of trauma narratives, considering the social function of individuals at the time of exposure and using objective measures of narrative characteristics through textometric analyses. More specifically, the aim of this study was to verify the presence of elements of control and social cohesion in resilient individuals, especially those with a professional and rescue social function.

To do so, we studied the narratives of traumatic experience of individuals exposed to the November 13, 2015, attacks in Paris and Saint-Denis, France. Participants were exposed in a civilian way (on the Parisian terraces, in the Bataclan, a concert hall or in the Stade de France, a sports stadium) or professionally (by being a first-aider, soldier, policeman or firefighter). We assume that this difference in traumatic exposure implies different social functions during the event, geared towards survival for civilians, and towards control, rescue, and protection for professionals. For our narrative analyses, we took account of 1) the development of PTSD, and 2) the social function during exposure. We constituted 4 groups of participants: civilians with PTSD, civilians without PTSD, professionals with PTSD, professionals without PTSD.

First, following the systematic review of Bedard-Gilligan & Zoellner (44), we expected that civilian’s narratives contain more perceptual, sensory, and central details related to the traumatic exposure than first-responder’s narratives, and that narratives of civilians with PTSD contain more singular first pronouns use, and more vocabulary related to dissociation and bodily injury or death. Then, following Gershuny et al. (33), Skogstad et al. (34) and Motreff et al. (35), we expected that first-responders’ narratives, compared to civilian’s narratives, contain more vocabulary related to stress preparation, and relief, protective and collective action. Finally, following Bonnano et al. (38) and Perrin et al. (36), we expected that the narratives of individuals without PTSD, compared to the narratives of individuals with PTSD (civilians or first-responders), contain more vocabulary related to control and action during the event, and fewer mentions of peritraumatic reactions (physical and emotional).

## Material and methods

### Participants

This study is based on the “Programme 13-Novembre” which studies the construction and evolution of the memory of the terrorist attacks in Paris and Saint-Denis on November 13, 2015 (45). This nationwide program was also described and investigated in Leone et al. (46), Mary et al. (47) and Postel et al. (48). Diagnostical measures were carried out 7-18 months after the attacks.

This program is based on two main studies, the “Study 1000” and the “REMEMBER study”. The nature of the participants ‘social function during the exposure (civil or professional) and narratives of the event were collected as part of the “Study 1000”. The presence of PTSD was diagnosed by experienced psychiatrists using the DSM-5 Structured Clinical Interview (SCID) as part of the “REMEMBER” study. All exposed participants met DSM-5 criterion A, indicating that they had experienced a traumatic event. We included subjects with full and partial PTSD among those with PTSD subjects. Partial PTSD is defined as having at least re-experiencing symptoms (criterion B) that persisted for more than one month (criterion F) and caused significant distress and functional impairment (criterion G) and were not due to another cause (criterion H).

The participants were divided into 4 groups according to their social function and the presence of PTSD: 1) Civilians exposed to these attacks who developed PTSD (group named CV_PTSD+), 2) Civilians who did not develop PTSD (named CV_PTSD-), 3) First responders involved during these attacks who developed PTSD (named FR_PTSD+), 4) First responders who did not develop PTSD (named FR_PTSD-).

A total of 59 participants were included in this study. Among these 59 participants, 37 are civilian exposed to the terrorist attacks (22 CV_PTSD+; 15 CV_PTSD-) and 22 are first responders during the attacks (5 FR_PTSD+; 17 FR_PTSD-). Details of the participants’ exposure are presented in **Table 1**.

**Table 1:** Details of exposure areas for civilians and job specificity for first responders.

Group/Exposure details	CV_PTSD+	CV_PTSD-	FR_PTSD+	FR_PTSD-
Bataclan (concert hall) Parisian terraces Stade de France Multiple sites Total	19 1 2 / 22	12 2 1 / 15	2 0 2 1 5	8 2 3 4 17
Profession				
Police officer Fireman First rescuer Security agent			3 0 0 2	11 2 4 0

CV_PTSD+, civilians with PTSD; CV_PTSD-, civilians without PTSD; FR_PTSD+, first responders with PTSD; FR_PTSD-, first responders without PTSD.

Participants were between the ages of 18 and 60 and French-speaking. They were asked not to use psychostimulants, drugs, or alcohol before and during the measurement sessions. If this was the case, the participants were excluded from the study. Written informed consent was obtained from all subjects.

Non-parametric analyses of variance and Chi-squared test were conducted to examine patient demographic characteristics between the experimental groups (**Table 2**). Gender was equally distributed among CV_PTSD+, CV_PTSD-, FR_PTSD+, FR_PTSD-, χ^2^ (3) = 1.98, p = 0.576. Variance analysis performed on age showed no general effect of group, χ ^2^(3) = 7.47, p = 0.058, nor significant *post-hoc* analysis between the 4 groups. There was no significant general effect of group in level of education, χ^2^ (21) = 20.70, p = 0.477.

**Table 2:** Participants demographic and clinical characteristics.

Group	CV_PTSD+	CV_PTSD-	FR_PTSD+	FR_PTSD-
N (Women)	22 (11)	15 (5)	5 (2)	17 (5)
Age in years, *mean (SD)*	33.86 (6)	38.09 (6.16)	44.76 (9.5)	36.62 (6.28)
Education level in years, *mean (SD)*	7.95 (1.68)	8 (1.51)	5.2 (2)	7 (2.12)

## Measures and procedure

### Assessments of the narrative of the traumatic experience

The narratives of the experience of the attacks of 13.11.15 in Paris and Saint-Denis were obtained within the framework of “Study 1000” of the “Programme 13-Novembre” (45). In this study, mediators, investigators, and researchers collect and analyze the testimonies of a group of 1000 volunteers, during four open-ended filmed interview campaigns spread over 10 years. The duration of the interviews varies and mobilizes the volunteers for up to half a day at the filming location. An interview guide contains the questions, the reminders (*relances*) and the instructions. The recordings are made by the *Institut national de l’audiovisuel* (INA) and the *Établissement de Communication et de Production Audiovisuelle de la Défense* (ECPAD). To transcribe the narratives, the audio track of each interview was extracted from the images, transcribed in speech-to-text format, then listened to again and corrected manually. The interviews were pseudonymized and conducted between 7 months and 17 months after the events of November 13, 2015, according to participants.

The narrative segment used in this study concerns the first part of the interview, in which the participant is asked to answer the question “*Can you tell me about November 13, 2015*”. Based on previous research, the beginning of the analyzed narrative segment has been defined as the first expression of danger and the end as the expression of the end of danger (49). More broadly, the end of the discourse is marked by a shift to a different spatiotemporal context than the November 13, 2015.

### Textometry analyses

Narrative analyses were conducted using the textometry software *IRAMUTEQ* (*Interface de R pour les Analyses Multidimensionnelles de Textes et de Questionnaires*). Iramuteq is an automatic text analysis software. It is based on the Alceste method, on R statistical software and uses the Python language. Before proceeding with the textometric analyses and to be able to import the corpus into the software, we corrected the corpus (checking spelling and grammar) and harmonized the terms used (terms referring to the same idea or object). After lemmatization, i.e. a technique enabling different forms of the same word to be reduced to a single form, only full words (nouns, adjectives, verbs, adverbs) were retained for analysis.

We used three complementary statistical methods to process textometry analysis:

- **Specificity analysis**: this method uses relative frequencies, i.e., the frequency of a word’s appearance divided by the size of the corpus portion. The specificity model (50) calculates, for each word and each part of the corpus, an index measuring the strength or weakness of the word in each part. A negative index represents under-representation of the word in the part, while a positive index represents over-representation. Values closest to -2 and +2 are not considered significant. A specific index of over- or under-representation is defined as a value exceeding ±2. Specificity analyses were used in this study to measure the words or word classes statistically under- or over-used by each experimental group, while considering the distribution of these in the corpus common to all 4 groups. The calculation of specificity cannot be used to draw initial interpretations and conclusions about the content of a corpus. However, it enables initial observations concerning the lexical uses expressed by each experimental group.
- **Top-down hierarchical classification using the Reinert method:** The transcriptions were subjected to a Descendant Hierarchical Classification (DHC) in the *IRAMUTEQ* software. The software searches for patterns of co-occurrence of words through successive Chi^2^ tests and organizes themes/clusters based on them. The Reinert method splits the text into segments, then observes the distribution of full forms in each segment. Then, it groups the segments into classes according to the words that compose them by a process of iteration. The Reinert method produces stable classifications. The software settings chosen to perform the analysis are as follows:

  - Number of final classes (Indicates the number of final classes in the classification) = 10
  - Minimum number of text segments per class (determines a minimum threshold of text segments below which the classes will not be retained, 0 is automatic) = 0
  - Minimum frequency of a form analyzed = set strictly greater to 2, according to Reinert method.

The introduction of illustrative variables (in this case, our 4 groups) into the corpus implemented in *IRAMUTEQ* then enables us to measure the lexical place occupied by each group in the results of the hierarchical classifications. In this case, *IRAMUTEQ* calculates the link between each word (or class of words) and the variable using a Chi^2^ test.

- **Correspondence Analysis (CA):** CA is an exploratory technique that allows to graph frequencies by crossing two or more qualitative variables. CA can be used to summarize and visualize the information obtained from the DHC. However, its interpretation remains the responsibility of the experimenter, who interprets the results of the CA according to his or her knowledge of the corpus.

## Results

### Textometric analysis of the narratives

Textual corpus included the transcribed testimonies of 59 participants, providing a complete corpus of 268,798 occurrences (words). We ran non-parametric analyses of variance (ANOVAs) and Mann-Whitney U tests to compare the length of the narratives. Narrative length (number of words) did not differ significantly between the four groups, χ^2^ (3) = 3.74, p = 0.291, between civilians and responders (p= 0.102), nor between PTSD+ and PTSD- (p = 0.503) (**Table 3**).

**Table 3:** Meanlength of narratives per group.

	CV_PTSD+	CV_PTSD-	FR_PTSD+	FR_PTSD-
Mean length of narratives in words (standard deviation)	4728 (2735)	4586 (2340)	2589 (1095)	3806 (2544)

### Specificity analyses

Orthographic lemmatization was carried out before calculating specificities to obtain statistically and semantically more accurate and significative measurements. The 10 most specific words for each experimental group are shown in **Table 4**.

**Table 4:** Results of the specificity analysis: presentation of the 10 most specific words in the narratives of each sub-group.

	CV_PTSD+	CV_PTSD-	FR_PTSD+	FR_PTSD-
CV_PTSD+ specificities				
I Finally Me What He Really Mostefaï Concert Completely Corinne*	45.251 33.26 26.78 16.54 12.48 11.59 10.83 10.05 9.04 8.12	0.48 -0.85 -0.79 -3.35 -1.53 -2.24 -3.97 2.53 -3.78 -2.98	-0.912 -4.64 -2.60 2.67 -1.08 -0.88 -0.65 -4.26 -0.45 -0.48	-61.25 -30.32 -24.40 -15.95 -7.82 -6.16 -3.65 -25.54 -3.00 -2.73
CV_PTSD- specificities				
Thus Paul* Matthieu* Solène* SMS She Have Open Taxi Cop	-23.79 -6.88 -3.49 -4.36 -4.06 1.97 -0.93 -1.79 -0.62 -1.61	18.58 16.65 11.28 10.55 10.36 9.69 8.53 8.30 8.26 7.46	3.85 -0.69 -0.67 -0.438 -0.30 -3.38 -0.67 -0.85 -1.36 -0.34	3.92 -3.91 -3.78 -2.48 -0.39 -16.01 -5.02 -3.26 -7.69 -4.05
FR_PTSD+ specificities				
Colleague Grating Job Personal Habit There Command post (cp) France Stadium Explosion	-27.98 -2.29 -2.15 -3.21 -1.26 -1.63 -2.60 -2.14 -0.40 -1.30	-20.55 -0.73 -2.47 -1.77 -0.38 -0.79 -0.51 -5.47 -7.00 -1.12	16.54 11.00 10.54 9.44 9.23 9.23 9.11 8.28 7.18 6.45	35.33 -1.30 0.58 0.43 -1.93 -0.31 -0.70 3.17 1.57 0.35
FR_PTSD- specificities				
Victim Vehicle We Colleague On Chef Truck Intervention Team Facility	-21.07 -24.88 -22.57 -27.98 -13.71 -8.73 -6.55 -9.31 -8.83 -6.65	-15.07 -8.77 -1.99 -20.55 -3.73 -7.80 -6.31 -6.18 -8.48 -4.11	-1.76 -0.65 0.86 16.54 -0.70 0.36 -0.79 0.53 2.50 -0.67	58.74 48.28 35.43 35.32 32.27 22.27 20.73 19.67 17.34 17.00

*First names have been changed to preserve anonymity.

Specificity analyses show that CV_PTSD+ overuse the first-person singular pronoun and words related to comprehension and interpretation of the attacks. CV_PTSD- overuse the first names of other people (usually other victims) and words related to the spatio-temporal context of the attacks. FR_PTSD+ overuse words of professional work habit, hierarchy, and the spatio-temporal context of attacks. FR_PTSD- overuse words associated with colleagues, helping others, professional intervention, and safety.

### Descendant hierarchical classifications

**Figure 1:**
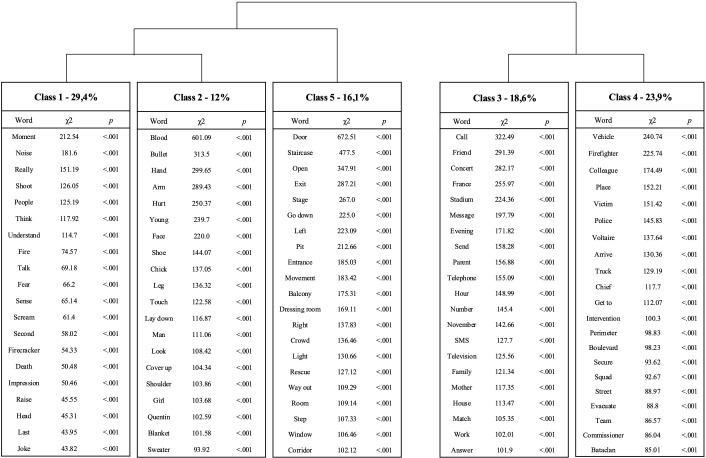
Dendrogram of the 20 words with highest χ2 in each lexical classes of the DHC.

Among the 7704-text segmentation of the first corpus, 98.87% were retained out of the total (=7617 segments classified out of 7704) and organized in 5 classes (**Figure 1**). Firstly, the software split the corpus in two sub-corpora:

1. **Central elements of the terrorist attacks** composed by Class 1, 2 and 5. *IRAMUTEQ* software also split this sub-corpus again, opposing Class 5 to Class 1 and 2.
2. **Peripheral context of the terrorist attacks** composed by Class 3 and 4.

**1. Central elements of the terrorist attacks** (Class 1, 2 and 5).

**Class 1 named** “**Mutual aid and personal injury**” was equivalent to 12% of the text segmentation. The main elements related to this class were: “*blood, bullet, hand, arm, hurt, young, face, shoe, chick, leg, touch, lie down, man, gaze, cover, shoulder, flesh, remove*”, among others. This Class 1 contains elements specific to the body and bodily injury, as well as elements of self-help action and an integration of others (first names of other victims, “woman,” “man,” “chick,” “guy”). The following extracts illustrate this content: “*We were covered in blood, on our clothes, on our shoes, on our sweater, on our coat, so that’s when the horror really hit home.*” “*We also helped a lady who had been shot in the right flank, who didn’t seem to be too badly injured*”.

**Class 2 named** “**Emotional and sensory-perceptual details**” was equivalent to 29.4% of the text segmentation. The main elements related to this class were: “*moment, noise, really, shoot, people, think, understand, fire, speak, fear, sense, scream, second, firecracker, death, inkling”*, among others. This Class 2 contains emotional, sensory, and perceptual details related to exposure to and understanding of extreme violence or death. The following extracts illustrate this content: “*And then everyone was running, the noise, the smells, the smoke, you could tell there was something, and people were screaming*”. “*And I remember all the anguish and the horror, the fear that I had held in, that I had kept inside me like that*”.

**Class 5 named** “**Escape and survival**” was equivalent to 16.1% of the text segmentation. The main elements related to this class were: “*door, floor, open, exit, stage, go down, left, pit, entrance, movement, balcony, lodge, right, crowd, light, rescue, room*”, among others. This Class 5 contains elements specific to the context of confinement related to the attacks of 13.11 including the “Bataclan” theater, and survival content (possibilities and details of escape). The following extracts illustrate this content: “*I have a very precise perception of this run of a few meters between the pit and the stage, then between the stage and the exit, then the whole run behind, outside*”. “*So up to the exit, up to the first emergency exit, first swinging doors, first swinging doors that were both open, I managed not to step on people*”.

2. **Peripheral context of the terrorist attacks** (Class 3 and 4).

**Class 3 named** “**Social and familial contacts**” was equivalent to 18.6% of the text segmentation. The main elements related to this class were: “*call, friend, concert, France, stadium, message, evening, send, parent, telephone, hour, number, November, SMS, tv, family, mother*”, among others. This Class 3 contains elements dependent of the family, social and societal setting of the attacks. The following extracts illustrate this content: “*So I was absolutely not serene, I was not well at all, I tried to call all my friends, people, close people in fact, who was in Paris or at the Stade de France*”. “*I immediately called my wife, and the first reaction all my guys had was to call their families*”.

**Class 4 named** “**Professional intervention**” was equivalent to 23.9% of the text segmentation. The main elements related to this class were: “*vehicle, firefighter, colleague, place, victim, police, Voltaire, arrive, truck, chef, give back, intervention, perimeter, boulevard, secure, BRI, street*”, among others. This Class 4 contains elements specific to professional actions of protection, rescue, and intervention with civilian populations and victims of terrorist attacks. The following extracts illustrate this content: “*I stayed a few minutes behind the colleagues who were on the corner of the Passage Amelot in an assault column, knowing that one of the terrorists had a shotgun.*” “*On the sidewalk, after the incident, my teams checked and classified the condition of the victims and gave them first aid*”.

**Figure 2:**
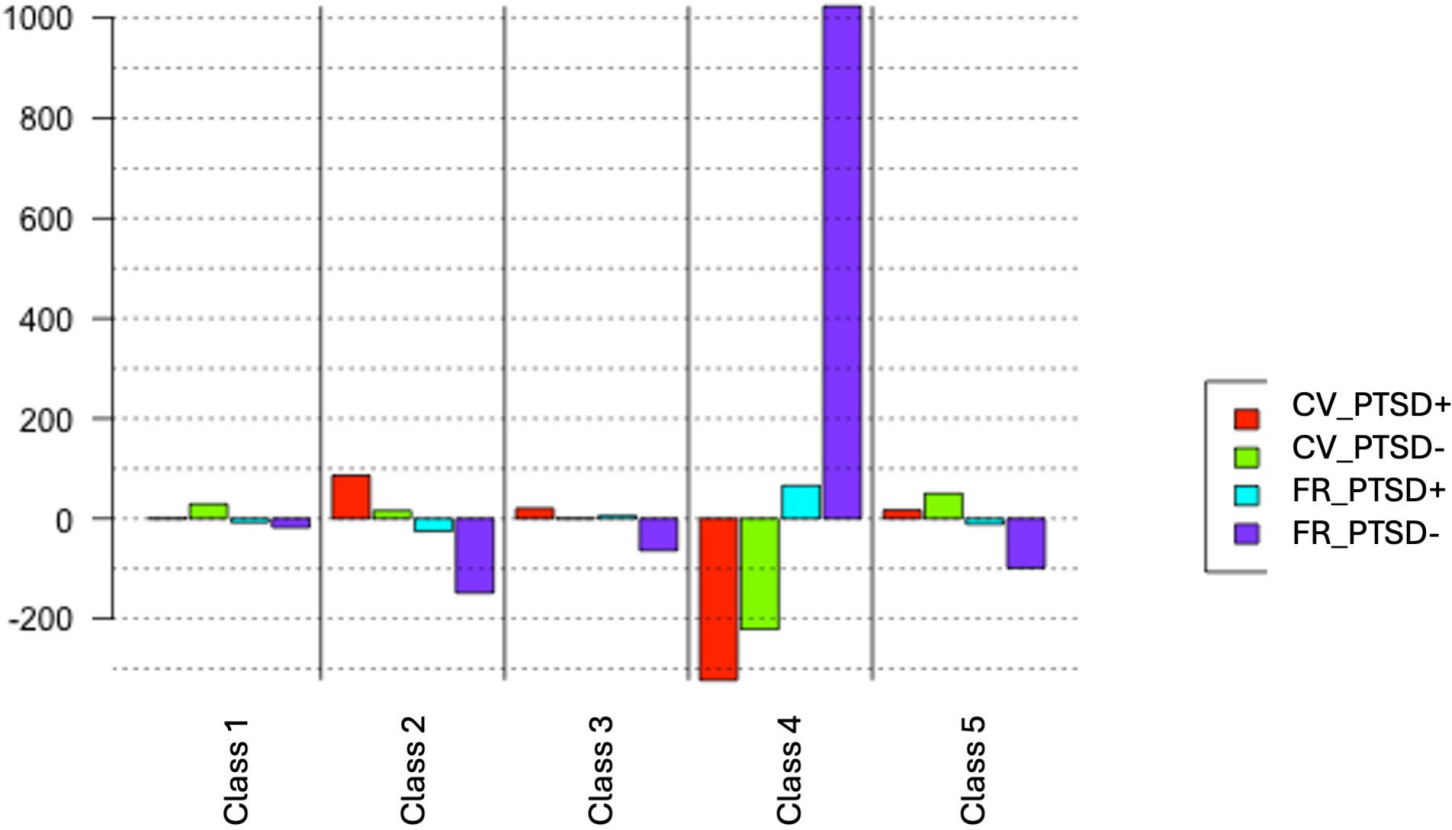
projection of the “group” variable on the classes from the DHC. CV_PTSD+, civilians with PTSD; CV_PTSD-, civilians without PTSD; FR_PTSD+, first responders with PTSD; FR_PTSD-, first responders without PTSD. Class 1, Mutual aid and personal injury; Class 2, Emotional and sensory-perceptual details; Class 3, Social and family contexts, Class 4, Professional intervention; Class 5, Escape and survival.

The illustrative group variable (CV_PTSD+, CV_PTSD-, FR_PTSD+, FR_PTSD-) allow us to project the over- or under-representation of each group in the five lexical classes (**Figure 2**). In terms of occupation of lexical space, we measured a difference in the importance of specific vocabulary according to their social function during exposure and PTSD:

- **Class 1 “Mutual aid and personal injury”** was solely illustrated by CV_PTSD- group, indicating that exposed individuals without PTSD are the ones contributing the most to this vocabulary in the corpus.
- **Class 2** “**Emotional and sensory-perceptual details**” was illustrated by CV_PTSD+ and CV_PTSD- groups, indicating that civilians are the ones contributing the most to this vocabulary in the corpus.
- **Class 5** “**Escape and survival**” was illustrated by CV_PTSD+ and CV_PTSD- groups, indicating that civilians are contributing the most to this vocabulary in the corpus.
- **Class 4 “Professional intervention”** was illustrated by FR_PTSD+ and FR_PTSD- groups, indicating that professional exposed individuals are the ones contributing the most to this vocabulary in the corpus.
- **Class 3 “Social and family contacts”** was illustrated by CV_PTSD+ and FR_PTSD+, indicating that exposed subjects with PTSD (civilians and first-responders) are the ones contributing the most to this vocabulary in the corpus.

### Correspondence analysis

The CA identified two factors that explained the five classes. The first factor, explaining 32.84% of the corpus information, corresponds to the social function of the individuals during exposure to the traumatic event (civil vs professional). The second factor, explaining 25.20% of the corpus information, corresponds to the degree of post-traumatic symptoms (with or without PTSD).

## Discussion

### Summary of findings

The aim of this study was to measure differences in content of trauma narratives of civilian and first-responders exposed to the same traumatic event (the November 13, 2015, terrorist attacks in Paris and Saint-Denis) with or without PTSD, according to their social function during exposure.

This study highlights a common traumatic memory built around 5 major lexical categories: “*Mutual aid and personal injury*” “*Emotional and sensory-perceptual details*”, “*Escape and survival*”, “*Professional intervention*”, “*Social and familial contacts”.* Civilians or first-responders with or without PTSD, however, seem to contribute differently to this narrative.

First, our results confirm that narratives of traumatic experience differ according to the social function during the traumatic exposure and its intrinsic characteristics. Civilians’ narratives with or without PTSD contain common sensory and perceptual elements, which are linked to the central context of the traumatic event. Direct exposure to the traumatic event and its most aversive details have certainly influenced the integration of these cues into the narratives. First responders’ narratives are made up of elements related to intervention, rescue, and protection of exposed civilians, as well as professional and hierarchical missions.

Secondly, our results show a difference in the narratives of the traumatic event according to the presence of PTSD. Specifically, we find that civilians and first responders suffering from PTSD recount their experience of the event with more details related to social and family contacts during the traumatic experience than those without PTSD. In comparison, civilians and first responders without PTSD recount their experiences of the event with more elements of peritraumatic collective action (rescue, collective support, bodily injury, death-related details, mutual aid).

We interpret these results by considering two factors: the presence of peritraumatic reaction cues in trauma narratives, and the presence of individual or collective control exercised during the event.

### Interpretation of data

#### Peritraumatic reactions and social context in narratives

Contrary to our hypotheses, civilians with PTSD and civilians without PTSD evoke the same amount of perceptual, sensory, and emotional details in their narrative of the traumatic experience. Details such as “*the smell of gunpowder*” or “*the sound of firecrackers*” are proposed in the narratives of both groups. This result runs counter to previous studies showing an over-representation of trauma-related perceptual and sensory details in the narratives of individuals with PTSD compared to individuals without PTSD (17, 18, 25). It is possible that these perceptual, sensory, and emotional details of the event correspond to the intrinsic reality of the peritraumatic experience of exposed civilians, which over time becomes part of the fixed and constructed details of the history of the event. Furthermore, the November 13, 2015, attacks in France impacted thousands of people (direct victims, witnesses, relatives, professionals, or citizens). Because of the collective nature of the traumatic event, individual memory could not be constructed without the influence of collective memory. According to Maurice Halbwachs (cited by 37) it is a group’s shared beliefs and collective experiences that shape the meaning of individual memories, and not the other way round. Thus, “*the act of remembering is essentially a social act, insofar as memories lie at the interface of personal identity and collective representations*” (51). In the wake of the November 13, 2015, attacks, the direct victims have for the most part narrated the event multiple times, in various contexts (judicial, medical, therapeutic, or other). These individual recollections of the event, combined with the recognition and societal dissemination of images and testimonies of the event, may have contribute to the collective memorization of characteristic details. Further study of these sensory and perceptual cues in narratives of a collective event could help refine our result.

Consistent with our hypothesis, civilians with PTSD use the first-person singular pronoun more often than civilians without PTSD and first responders with and without PTSD. Higher first-person singular pronoun use may indicate a self-centered view of the traumatic experience, with little inclusion of other people and the external context of the event. Prior research showed that the use of first singular pronouns in trauma narratives may represent a lack of psychological distancing (52) while the distancing of elements from the past is shown using indeterminate pronouns (53). Chung and Pennebaker (54) point out, based on past literature, that excessive use of the first-person singular pronoun is associated with negative affective states.

Civilians without PTSD mostly mention the names of other victims, while first responders with and without PTSD are more likely to mention colleagues and their intervention team. Studies showed that individuals who use more first-personal plural pronouns and collective mentions in trauma narratives had fewer PTSD symptoms (25). The mention of the collective in narratives could represent a social dynamic of sharing the experience with others (55). The use of the first person plural pronoun “we” may then imply a sense of group identity (56). We discuss the social characteristics of traumatic exposure in civilians in the second section of this discussion.

Civilians without PTSD also use more words related to death and injury than civilians with PTSD, such as “*blood*”, “*bullet*”, “*wound*”, “*flesh*” and “*body*”. This finding is contrary to previous studies showing an overuse of death-related words in exposed individuals who have developed chronic PTSD (57, 58). Indeed, these studies indicated that chronic PTSD and memory intrusions would result in strong recall of trauma-related “hotspots” in narratives, i.e., the most emotional and intense elements of the memory. The fact that the PTSD civilians in this study did not participate in this vocabulary may indicate experiential avoidance, a mechanism frequently described in PTSD (59) and defined as an unwillingness to remain in contact with aversive private experiences (i.e., bodily sensations, emotions, thoughts, behavioral predispositions). D’Andrea et al. (25) found that exposed individuals who used fewer anxiety-related words reported more long-term post-traumatic symptoms. In the present study, civilians who have developed PTSD and do not evoke negative details in their narratives may exhibit experiential avoidance with a persistent inability or refusal to address painful affects related to the experience (60).

Civilians and first-responders with PTSD evoke the social and family context associated with the November 13, 2015, attacks. Numerous studies have shown the importance of the social context in traumatic exposure and the development of PTSD, especially when the exposure was collective (61). The mention of this lexicon by civilians and first responders with PTSD could illustrate a strong emotion felt towards loved ones during the event, and *a posteriori* potentially exacerbated by the presence of the disorder. In civilians with PTSD, this emotion is found in the concern expressed for the safety of loved ones or family, or in the concern expressed by their distant loved ones through phone calls or text messages during the event. Previous studies have shown that worry for others during a traumatic event is a significant PTSD risk factor with a negative influence on post-traumatic adjustment (62, 63). In first responders with PTSD, this emotion is relative to their own family, or to the victim’s loved ones. While the former might reflect a potential breach of the emotional distancing related to their own lives required in first responders, the latter might illustrate excessive empathy towards victims. Many professionals with PTSD reported that they immediately thought of the loved ones of the deceased victims and the repercussions of this death on their future lives. However, once again, due to the *a posteriori* recollection of the traumatic memory, we cannot know whether this mention of family and victims in first responders with PTSD is a cause or a consequence of the disorder. An assessment of the presence of personality cues of compassion and empathy for victims prior to traumatic exposure would help answer this question.

#### Individual and collective control in narratives

Several studies have measured the use of the first-person singular pronoun in PTSD narratives to measure self-referential perspectives. However, as O’Kearney et al. (18) point out, none have directly measured self-referential perspectives in narratives through themes of sense of control. In line with our initial hypotheses, our study shows that exposed individuals without PTSD (civilians and first-responders) include more elements of control in their narratives than exposed ones with PTSD. However, this self-referential is different in civilians without PTSD and in first responders without PTSD.

Civilians without PTSD evoke elements of mutual aid, collective action, and sense of control. Several studies suggest that, in the case of collective traumatic events, narratives that contain elements of collective emotion and action reveal a greater sense of social support, which helps limit the effects of stress on well-being (17). The “sense of belonging”, which corresponds to the feeling of being part of a group, of a whole (in this case, the trauma exposed community), is an important resilience factor in the context of collective traumatic exposure (64). However, our experimental methodology does not allow us to specify whether these mentions of collective control and mutual aid constitute a protective factor for PTSD, or whether its presence in the narratives is better explained by the absence of the disorder. The absence of characteristic symptoms of PTSD such as negative beliefs about oneself, others or the world, or heightened feelings of guilt or shame may also influence this content of collective control and mutual aid. Studies involving both pre-traumatic and post-traumatic observation methodology in individuals exposed to a traumatic event would help refine this finding.

First-responders without PTSD recount the traumatic event almost exclusively using words associated with the context of intervention, teamwork, and civilian protection. This mention of professional intervention is found both in the lexical specificities of their testimony (with the words “*victim, intervention, colleague, safety, team, we*”) and in their participation in the lexical field associated with intervention. On the other hand, the absence of lexical fields associated with central and contextual details of the event seems significant. Less attention and thus less encoding of these details of the traumatic event in trained professionals without PTSD could explain this non-integration of such details in the narratives. This result is consistent with our initial hypotheses and with the existing literature, suggesting that the preparation for stress and sense of control and social cohesion during the event may protect from developing PTSD.

For first responders with PTSD, intervention revolves mainly around the notion of professional hierarchy, with words like “*work, staff, habit, order, boss, driver, chief*”. Several studies have shown that professionals who shift action and responsibility onto authority figures, both in their professional lives and in their narratives, are at greater risk of acute dissociative responses to trauma and subsequent PTSD. The external locus of control, i.e., the generalized belief that the occurrence of events and their content do not depend on the individual’s own behavior but on external environmental factors such as fate, chance, or other people, is associated with greater severity of PTSD and psychological distress in professionals (65). This also demonstrates the importance of the collective in professional exposure, both in the actions taken during the event and in the image of the power exercised. Skogstad et al. (34) showed that exposed police officers had a high level of post-traumatic symptoms when they reported feeling mentally overwhelmed during the traumatic event. A narrative study of Backteman-Erlanson et al. (66) showed that during traumatic intervention, police officers may use control and action strategies based on past experiences, which promoted a sense of security. However, these strategies could sometimes be inadequate or insufficient in some traumatic exposure, leading some officers to feel overwhelmed. Narratives of first responders with PTSD can also be characterized by work idealization and work-related disillusionment (67) and feelings of guilt or shame (68, 69). Military professionals may also have great difficulty discussing a traumatic event and post-traumatic reactions due to significant professional and social consequences and potential stigmatization of mental disorder in both domains (70).

## Limitations and future directions

While we found promising results in the current study, some limits also merit discussion. First, the participants (especially first responders with PTSD) are comprised of a small sample of exposed individuals, and may, thus, not be generalizable. Secondly, this study investigates the narrative elements produced by exposed civilians or first responders with or without PTSD at a distance from the traumatic event. We must be cautious in interpreting results obtained from retrospective data of peritraumatic experience, as the accuracy and coherence of long-term traumatic memories are still controversial (71). Nonetheless, this study measures differences in traumatic narratives according to PTSD and their social function during the exposure, a methodology that was, to our knowledge, unprecedented. Another constraint comes from the specific time interval between the attacks and the narratives. This length of time may have led to changes in discourse, notably due to differences in disclosure possibilities, or to judicial, professional, medical, or therapeutic conditions that may have necessitated a narration of the event and shaped the memory. The time between such collective traumatic exposure and the narrative may also have facilitated the development of a common social language around the event or have favored the fixation of certain episodic details over time. Our interpretations must therefore consider the sociological effects of this traumatic experience, as studies have demonstrated a possible creation of a common post-traumatic sense of control among collective trauma survivors (72) and first responders (73). The presence of PTSD, or post-traumatic symptoms (feelings of shame or guilt) may also have influenced narratives. Self-reported assessments of post-traumatic cognitions, which can influence traumatic narratives (74) would have provided additional information. To confirm our findings, future studies should propose an assessment of the sense of pre-, peri- and post-traumatic control associated with the event to measure its influence on the narration of the traumatic event (75). Finally, further analyses using stronger tool with a large language model could help to confirm our results. The non-use of this type of tool is a limitation of this study.

## Conclusion

This study is unique in its aim to investigate the influence of the presence of PTSD and social function in the narratives of civilians and first-responders who have been exposed to the same collective traumatic event. We show that civilian exposure to the traumatic event influences content related to dissociation and peritraumatic emotion in narratives. In contrast, the narratives of first responders, considered more prepared for this type of event, contain more peripheral and contextual details. We thus show that the stress preparation of first responders can limit the encoding and recall of perceptual, sensory, and emotional elements of the traumatic event. Future studies could verify this influence of the role played during the traumatic exposure by targeting a better understanding of the coping mechanisms of action or emotional regulation according to the civilian or professional role. This study also shows that non-PTSD individuals produce narratives characterized by individual and collective control. These findings add to a growing body of research and extend previous research on trauma narratives. Our study measures the elements of traumatic memory that are important enough for the individuals interviewed to be given priority in their traumatic narratives. More studies are needed to replicate our findings and further examine the relationship between traumatic narratives and long-term post-traumatic adaptation.

## Data Availability

The raw data supporting the conclusions of this article will be made available by the authors, without undue reservation.
